# A Control Process for Active Solar-Tracking Systems for Photovoltaic Technology and the Circuit Layout Necessary for the Implementation of the Method

**DOI:** 10.3390/s22072564

**Published:** 2022-03-27

**Authors:** Henrik Zsiborács, Gábor Pintér, András Vincze, Nóra Hegedűsné Baranyai

**Affiliations:** Renewable Energy Research Group, Soós Ernő Research and Development Center, University Center for Circular Economy, University of Pannonia Nagykanizsa, 8800 Nagykanizsa, Hungary; pinter.gabor@uni-pen.hu (G.P.); vincze.andras@uni-pen.hu (A.V.); baranyai.nora@uni-pen.hu (N.H.B.)

**Keywords:** solar energy, active tracking system, sun tracking, sun-tracking sensor, azimuth angle, tilt angle

## Abstract

What basically determines how much energy is generated by a photovoltaic (PV) system is the amount of solar irradiation that is absorbed by its PV modules. One of the technical solutions to boost this quantity, and thusly also maximize the return on PV investments, is solar tracking, which makes the following of the sun on its daily and annual journey in the sky possible and also takes changes in cloud conditions into consideration. The solar-tracking solutions that PV systems are most frequently equipped with deploy active sensor technologies, while passive ones are less common in present-day practice. However, even the popular solutions of today have their limitations. Their active sensor-tracking algorithms leave room for improvement for at least three major reasons, as they do not prevent the unnecessary operation of the motors in cloudy weather, they do not make the modules assume an appropriate position after nightfall, and they do not make sure that the structure and the electronics of the PV systems are protected from rain and the strong winds in the event of storms. This paper introduces a new active sensor-tracking algorithm, which has not only been tested but it is also in the process of patenting (patent ID: p2100209). By their contribution, the authors endeavor to propose a solution that can solve all three of the issues mentioned above. The concept is based on two fundamental findings. According to the first one, periodic movement can not only considerably decrease motor movement but also increase system lifetime, while the second one simply suggests that moving the modules into an almost horizontal position facing the equator at low light levels is conducive to the prevention of damages caused by storms and fast reaction to the increase in the amount of light at daybreak. A positive feature of the new system for PV power plant operators is that it performs the tracking of the sun practically without any decrease in power compared to the focal point position, since it works with an average inaccuracy of 1.9°.

## 1. Introduction

### 1.1. The Global Significance of Photovoltaic Technology

One of the greatest challenges facing humanity today is the phenomenon of climate change. The body of scientific knowledge related to its far-reaching and intertwined environmental, economic, and social impacts is growing day by day. Currently, the need to understand its negative consequences is gaining more and more attention worldwide [1,2,3], while the extent of these detrimental effects is constantly the focus of heated debates. Globally, targets have been set to significantly reduce the emissions of greenhouse gases and to keep the rise in global average temperatures under 2 °C in the coming years [4,5,6].

The constant advancement of technology and development in the world keep energy consumption and demand rising endlessly [7]. This growing trend in the face of the finite nature of fossil fuel supplies, coupled with the environmental problems associated with their use, calls for a transition from the ‘old’ sources of energy to less conventional ones. [8]. This means that a variety of renewable sources of energy, including solar, hydro, wind, biomass, etc., causing no or much less environmental pollution, are expected to take the place of the currently mainstream energy sources [9,10].

As the one existing in the greatest abundance, solar energy is the most promising, since it is considered to be not only clean but also essentially infinite [11]. Solar energy is also among the most widely used renewable sources of energy [12] thanks to its practicality, as solar radiation can be converted into both thermal or electric energy by the use of suitable technology, such as solar thermal converters or PV systems. Thus, it is little wonder that the spread of the latter in the world reached approximately 760 GW in 2020 [7]. In 2020, about 20 nations installed a total of more than 1 GW of new PV capacity each, with 14 of them now possessing a total installed capacity in excess of 10 GW each, with five of them having even more than four times that. After the European Union’s (EU) domination of the list for years until 2015, in 2020, the world leaders in terms of installed PV capacity were China (253.4 GW), the EU (151.3 GW), the USA (93.2 GW), and Japan (71.4 GW). The ranking of the 11 countries (including the EU) with the highest cumulative installed capacities seems to be more stable, however, thanks to substantial amounts of capacities installed previously. At the end of 2020, the following countries/unions were in possession of the largest total installed capacities:

- China: 253.4 GW;
- The European Union: 151.3 GW;
- The United States: 93.2 GW;
- Japan: 71.4 GW;
- Germany: 53.9 GW;
- India: 47.4 GW;
- Italy: 21.7 GW;
- Australia: 20.2 GW;
- Vietnam: 16.4 GW;
- South Korea: 15.9 GW;
- The United Kingdom: 13.5 GW [13].

As for the future, the nominal power of PV systems installed globally is forecasted to reach 1043–1610 GW by 2023 [14].

### 1.2. The Importance of Solar-Tracking Solutions in PV Technology

What primarily determines the output power of any PV system is the incident solar irradiance, the intensity of which is a function of a number of factors, including the tilt and azimuth angles of the solar module and the position of the sun [15]. Consequently, PV systems have to be designed [16] in a way that ensures the best possible PV module orientation and tilt angle for optimal operation. Otherwise, the power plant cannot generate the amount of electric energy that it could under ideal circumstances, which may adversely affect the economic indicators of the investment, e.g., by a longer payback period. Without being aware of the individual local climatic and other conditions (e.g., terrain) of the given site, it is not possible to maximize the quantity of energy produced, since the ideal settings (e.g., row spacing) can greatly vary between various locations [17,18]. A good example is the fact that the optimal tilt angle in the case of south-facing PV modules in Europe ranges from 20° to 50° [19].

While previously, mostly static or fixed PV systems were used, new technological developments allow the enhancement of the efficiency of today’s PV power plants by the use of solar-tracking systems, tracking the position of the sun all through the day and all through the year [16,17,20,21]. One of the benefits of installing PV systems of several GW in a given area is its easier technical management compared to scattered systems. As a consequence, the proportion of ground-mounted systems in 2018 was around 70%, which is expected to increase even further in the future [14,22]. In 2019, approximately 3–5% of all ground-mounted PV systems was equipped with solar-tracking technology, so it is an ambitious goal for this proportion to reach 10–15% within the next decade [14,23].

Although the use of solar-tracking systems enhances the efficiency of energy generation, and thus optimizes the whole process, it is achieved at a certain cost, as these systems are less resistant to unfavorable weather conditions and also require more preparation of the site, including extra trenching and grading [9]. Furthermore, the extra parts needed for such systems themselves lead to additional expenses too, which means that the growth in yield reached by the use of tracking must exceed the extra costs thusly incurred if solar tracking is to be viable in economic terms [24]. The country-specific economic aspects of solar-tracking systems were explored by Vaziri Rad et al., who pointed out in 2020 that the energy costs of solar-tracking systems deploying different technological solutions showed great variation [25].

According to their main two types, solar-tracking systems are either active or passive [17,26,27]. Passive systems work on the basis of the thermal expansion of some material with a low boiling point, which creates an imbalance that triggers the given mechanism. However, such solutions are not commonly used, as they are not too efficient despite their great complexity [8]. Active sun-tracking systems can be divided into further subgroups according to their driving methods and operational logic. Apart from controlling them manually, solar-tracking systems can also be controlled by a number of other driver systems [27]. In all these systems, it is the control signal that controls the direction and magnitude of the tracking action by providing the motor and the gears with the appropriate information. The following is a list of available active solar-tracking solutions for PV systems:

- Adaptive Neural Fuzzy Inference Systems (ANFIS);
- Digital Signal Microcontrollers;
- Electro-Optical Sensors;
- Fuzzy Logic;
- Fuzzy Neural Networks (FNNs);
- Fuzzy Rules Emulated Network (FREN);
- Light-Dependent Resistors (LDRs);
- Programmable Interface Controller (PIC) Microcontroller;
- LDR + ATmega;
- LDR + PIC Microcontroller;
- LDR Microcontroller;
- Light Intensity Sensors;
- Neural Networks;
- Open Loop and Closed Loop Systems;
- Rockwell Automation [27].

Based on their freedom of movement, active solar-tracking systems have two more categories: systems with one and two axes [28]. Single-axis systems track the sun’s movement in the sky from east to west, but their tilt angle remains the same during the whole process [29,30,31,32,33]. This means that the modules are not always at a right angle to the rays of the sun. This problem can be solved by dual-axis tracking, which can achieve both a higher irradiance intensity and conversion efficiency [17,34,35,36]. In such systems, apart from rotating from east to west, the modules are also moved in a north–south direction [9,26,37]. It seems to be a commonly accepted view held by many experts that solar tracking with devices with two axes can have higher efficiency and cost-effectiveness compared to single-axis ones when they are part of larger systems. Nevertheless, it must be carefully considered if the total costs of such equipment, also including the cost of the energy they need and their maintenance costs, are offset by the value of the extra electric energy produced with its help [17,38,39,40,41]. Comparing optimally positioned PV systems and single- and dual-axis tracking system solutions, it can be established that an annual surplus of generated energy of up to 20–45% can be reached by applying the latter [16,17,42,43].

A great amount of research has been carried out in the field of tracking system accuracy and efficiency. According to the results of Lee et al. (2009) [44], who examined many systems of high precision and the algorithms they used, the typical tracking error tolerance in those was between 0.0003° and 1°, while the expenses associated with such investments were rather substantial. It is, of course, also true that there are less expensive tracking systems as well, but their tracking error tolerance is 1.5° at least [45]. In the market, there are both simple, generic solar-tracking systems and customized products available [46]. The problem with the less sophisticated solutions is that their accuracy is lower, and they cannot spot the ideal position in terms of the maximum extractable power. Additionally, they do not move the modules into an optimal dawn position at sunset or sunrise, meaning that it is only done by the sensors with a considerable delay. These systems also tend to continuously seek the brightest spot of the sky, resulting in a constant search for direction when it is overcast. This unnecessary motion leads to the premature wear of the motors and all mechanical parts, while the amount of generated electrical energy is also adversely affected, as the superfluous motor operation consumes more energy. As for customized solutions, they are mainly made for concentrator photovoltaic (CPV) systems, which require continuous focusing. These tracking systems can also be used with concentrating solar power (CSP) systems. Although, unlike the simpler versions, these solutions offer accurate solar tracking, certain issues, such as those of the constant fine motor movement and cloud protection, have not been dealt with successfully yet [46].

### 1.3. The Characteristic of the Performance Insensitivity Thresholds

Nsengiyumva et al. (2018) came to the conclusion that an inaccuracy of 10° in solar tracking results in a 1.5% performance degradation in traditional crystalline PV technology regardless of orientation [20,47]. A 3-year series of measurements carried out in Hungary examined this problem in a complex context [46]. The research compared monocrystalline (m-Si), polycrystalline (p-Si), and amorphous silicon (a-Si) PV technologies with the help of a support system capable of dual-axis solar tracking. In the research, the direct radiation on the surface of the modules was set to an accuracy of 3 mm using the cells of a CPV module, the position of which was regarded as the focal point (FP). The main emphasis of the research was the change in performance depending on the deviation from the FP. To achieve the maximum power values of the PV modules, the experiment used a True Maximum Point Seeking (TMPS) artificial load, operating on oscillation principles, which was able to optimally adjust any power change within a second. With the help of this, by keeping the instantaneous power, i.e., the voltage multiplied by the current, at the highest value, the PV modules were always operating at the maximum power point. It was found that the power changes of the m-Si, p-Si, and a-Si modules were influenced by the magnitude of the deviation from the FP as well as by the direction of deviation and the cardinal direction. In the case of the crystalline PV modules, the changes in the cardinal directions influenced energy generation in the same way, which allowed the formulation of average values. Furthermore, up to a 10° deviation, the a-Si technology showed almost identical results to those of the crystalline version, but in the case of values above this, it was more sensitive to changes in the cardinal direction. In terms of performance changes, it was found to be most insensitive to north–south deviations, while it proved to be most sensitive to north–west, south–west, south–east, and north–east deviations [46].

The research also found that a 3° deviation from FP does not yet mean a detectable reduction in performance, regardless of the direction of the deviation or technology. This result can be used as a base for planning the accuracy of solar-tracking systems/sensors, for example, when it is considered from an economic perspective what the consequences of exceeding this 3° limit value in designing a sun-tracking device may be. Furthermore, the findings of the research can help with the design of m-Si, p-Si, and a-Si PV solar-tracking systems and with the calculations of the associated investment return indicators [46].

### 1.4. The Contribution of the Present Paper to Solar-Tracking Technologies

Active solar-tracking systems have many types and improvements. There are solutions that use different logics or artificial intelligence (AI) to track the sun with high accuracy, but the vast majority of these are still in the phase of testing or development. Other systems are based on astronomical or GPS-based solutions. Many approaches seek to solve the problem of precise solar tracking in some form, but they cannot handle environmental and weather conditions properly [27,48]. The vast majority of solutions available in the market use sensor-based, GPS, or other software solutions. The general disadvantage of solar-tracking PV solutions with active sensor driver system control is that the control procedures are inaccurate and have incomplete functions (e.g., cloud management and sensors with high sensitivity thresholds) [49].

The characteristics and five main shortcomings of the general control mechanisms of solar-tracking solutions based on today’s active sensor driver systems were explored by reviewing solar-tracking patents and technologies available in the global market. To the best of the authors’ knowledge, there is currently no active sensor-based solar-tracking method that does not have any serious deficiencies affecting energy production or maintenance requirements. The aim of the research presented herein is to present and verify a method of controlling solar-tracking PV systems, currently undergoing a patenting process (ID p2100209), based on the LDR microcontroller solution, which is able to eliminate the general shortcomings of active sensor driver system solutions by specific control mechanisms. The new procedure and the principles used to develop it can provide guidance for the further development of solar-tracking systems, while the practical application of the method directly contributes to increasing energy generation by solar-tracking systems and thereby improving their profitability. From the above, it also follows that the energy parameters of the system are not the subject of this study, as it is rather focused on the implementation of an algorithm for optimal control.

The structure of this paper is as follows: Section 2 presents an overview of the existing active solar-tracking systems and identifies their main deficiencies. Section 3 describes the experimental station used for the verification of the novel tracking system. Section 4 introduces the new algorithm in detail, while Section 5 discusses the results, with Section 6 presenting the conclusions drawn.

## 2. A Review of the Existing Active Solar-Tracking Systems

This study presents patents and technologies available in the global market that are relevant to its subject matter, based on their functional features. These are presented with a view to summarizing and comparing the solar-tracking solutions that apply sensor driver systems entirely or partly, as well as the solutions of the control mechanisms of the LDR microcontroller presented herein, whose specific features and potentials for application are easy to see with the help of this information. The following market products and patents were reviewed:

- FUSIONSEEKER DS-50D6W and FUSIONSEEKER DS-100D10 [50];
- ECO-WORTHY, dual-axis solar tracker controller [51];
- WST03-2 [52];
- Luoyang Longda Bearing Co., Ltd., solar-tracking controller [53];
- SunTura solar tracker [54];
- STA2000-HW [55];
- MLD sensor [56,57];
- WO 2020/185271 A1 patent description [58];
- EP 2 593 759 B1 patent description [59];
- WO 2013/074805 A1 patent description [60].

The reviewed market products and patents are described as follows:

- The Fusionseeker DS-50D6W, the FUSIONSEEKER DS-100D10 [50], and the ECO-WORTHY dual-axis solar tracker controller [51] control units use light-sensing sensors to search for the brightest spot in the sky. These solutions perform continuous sun tracking from sunrise to sunset, for which the sensitivity of the sensors to light can be adjusted. FUSIONSEEKER systems are characterized by the need for the user to set the light intensity value when sun tracking is prohibited, for example, due to cloudiness. In the case of the ECO Worthy solution, this feature is installed in a way that does not allow adjustment. In the case of Fusionseeker and ECO Worthy’s solutions, when there is some cloud cover but the light intensity still remains higher than the set value of the control unit, the search for the brightest point in the sky becomes continuous, and the probability that the motors start swaying unnecessarily increases. In other words, in the case of inadequate light intensity, the motors performing sun tracking due to the constant search will be subjected to increased wear and extra load due to the unnecessary operation. An advantageous feature of Fusionseeker is that with the help of a sensor, it can be set for sunrise. In addition, it also has strong wind and hail protection input ports, but these functions require the connection of an anemometer or hail sensor or a hail detection radar system. The device used by ECO Worthy is characterized by taking a horizontal position at night and in rainy weather or even in the case of strong winds if an anemometer is integrated in the system.
- The WST03-2 [52], the Luoyang Longda Bearing Co., Ltd., solar-tracking controller [53], and the SunTura solar tracker [54] control units perform continuous sun tracking from sunrise to sunset. The WST03-2 solution is capable of assuming a protective position in the case when an anemometer is integrated in the system. Luoyang Longda Bearing Co., Ltd.’s solution also has a protection function against strong winds when integrating an anemometer, and it is also capable of time-based control. One of the disadvantages of controlling by these products is that they are not equipped with cloud protection. Another less fortunate feature of these is that the control units hold the position reached at sunset and, therefore, the systems only turn towards the rising sun when the sun-tracking sensors are exposed to light of sufficient intensity. In addition, in the case of heterogeneous light conditions, e.g., cloudy skies, the search for the brightest point in the sky becomes continuous, and the likelihood of the unnecessary swaying motion of the motors increases. Thus, in heterogeneous light conditions, the solar-tracking motors work unnecessarily in these systems too.
- The STA2000-HW [55] technology performs continuous or delayed solar tracking with delays of 0–65 min. The time intervals need to be set manually. The system always looks for the brightest point in the sky; however, if the initial setting is not correct, the probability of unnecessary swaying by the motors increases. This may cause the increased wear of the solar-tracking motors. The system has wind protection, the operation of which is ensured by an anemometer, the sensitivity of which can be adjusted. The control keeps the position reached at sunset in a normal position, and this can be modified by the user.
- The control unit of the MLD sensor [56,57] performs continuous sun tracking from sunrise to sunset and strives to maximize the energy yield. As a result, in the case of heterogeneous light conditions, e.g., cloudy skies, the search for the brightest point in the sky becomes constant, thus increasing the likelihood of the unnecessary swaying motion of the motors. So, in heterogeneous light conditions, this system also has the problems seen above: unnecessary motor operation and increased wear. A favorable feature of the solution is that the sunrise position is set with the help of a sensor at dawn.
- In the case of patent description WO 2020/185271 A1 [58], the control unit enabling solar tracking performs continuous solar tracking using sensors and GPS data. In the event that the sensors are not exposed to sunlight, the continuous movement of the motors is achieved based on GPS data. The protection of the system against wind is made possible by sensors measuring wind speed and wind direction, with the help of which, these characteristics are continuously monitored. The solution adjusts an ever-changing protective position for stronger winds that minimizes damage to the system. The disadvantage of its application is that it is not protected against cloudiness, so when this solution is only controlled by sensors, due to non-uniform light conditions (e.g., cloudy skies), the search for the brightest point in the sky becomes continuous, and the probability of the unnecessary swaying of the motors increases, too.
- According to the solution presented in patent description EP 2 593 759 B1 [59], the position of the sun is continuously determined using seven sensors, which control three motors. Three sensors perform approximate orientation, while the three primary sensors together with signals from the central sensor control the motors. If the primary sensors and the central sensor are exposed to light of the same intensity, the control of the three engines will stop for as long as this condition persists. When there is some cloud but the light intensity still remains higher than the value set in the system, the search for the brightest point in the sky becomes continuous, and the probability of the unnecessary swaying of the motors increases. In other words, in the case of inadequate light intensity, the motors performing sun tracking due to the constant search will be subjected to increased wear and extra load due to the unnecessary operation. A feature of the solution is that at night, it assumes a horizontal position. The fact that three engines are required for operation can be considered disadvantageous.
- In the case of patent description WO 2013/074805 A1 [60], continuous sun tracking takes place. No other functions have been developed, and they must be added separately. The special feature of the solution lies in the fact that it allows the precise adjustment of direct sunlight.

The characteristics of the control mechanisms of the solar-tracking solutions using active sensor driver systems reviewed above are summarized in Table 1.

The active sensor-based solar-tracking control procedures used today for PV systems are basically not capable of accurately locating the sun. One of the observed characteristics is that the control unit does not put the PV modules in an ideal position after sunset or at sunrise (e.g., the PV system keeps the position reached at sunset), so the sensors that detect solar radiation can only do this with a significant delay, which negatively affects the daily electricity generation. The position maintained after sunset or the one facing eastwards are also unfavorable because, thusly, the mechanics are more exposed to the adverse effects of stronger winds, and the motor/electronics are exposed to the adverse effects of getting wet in the event of a storm. In this position, the mechanical parts practically act as a sail and, in the case of weaker mechanics, the supporting structure may be permanently damaged. For the vast majority of the solutions examined, a general control problem is the constant search for the brightest point in the sky, which causes a constant search for direction in cloudy weather. In technical terms, this means increased wear and extra load for the solar-tracking motors, and their unnecessary operation also results in less energy generated. In addition, when using overly sensitive sensors, the superfluous operation of the motors causes a continuous swaying motion in the sun-tracking mechanism, which diverts the PV panels from the ideal position, thereby reducing the energy that can be produced.

In summary, it can be stated that currently, there is no control mechanism available for the known devices using active sensor-based solutions that can solve the following five issues together:

- Periodic seeking for the brightest point in the sky;
- Preventing excess motor activity resulting from the search for direction due to cloudiness;
- Setting the system at sunset to a position that will be ideal at sunrise the next day;
- Significantly reducing the damaging effects of storms without an anemometer;
- Protecting the electronics and motors installed under the support structure from precipitation during a storm.

During the elaboration of the new solar-tracking procedure presented in Section 4, the main emphasis was placed on solving these five detected problems.

## 3. The Experimental Station Used in the Project

### 3.1. The Characteristics of the LDR Microcontroller

In the project, we used Arduino Nano microcontrollers, to which the LDR could be attached. The reason why this type of microcontroller was chosen for the realization of the procedure was that this device, which can be considered a complete circuit with a USB connection option, is relatively small in size, and it is compatible with the pin layout of dual in-line (DIL) integrated circuits. The USB connection option is an important feature because this allows the program to be loaded from a computer via a USB connection. Furthermore, the operational characteristics of the procedure operating at the solar power plant can be monitored, which means that it can be modified quickly, even on site, if necessary. In addition, a programming environment is also provided for microcontrollers free of charge. The newly developed procedure can be uploaded not only to the Arduino Nano, but also to the Arduino Uno, which is different in size, making it easier to use with control units of different sizes.

### 3.2. The Characteristics of the Measuring Station Used to Verify the Operation of the Procedure

**Figure 1:**
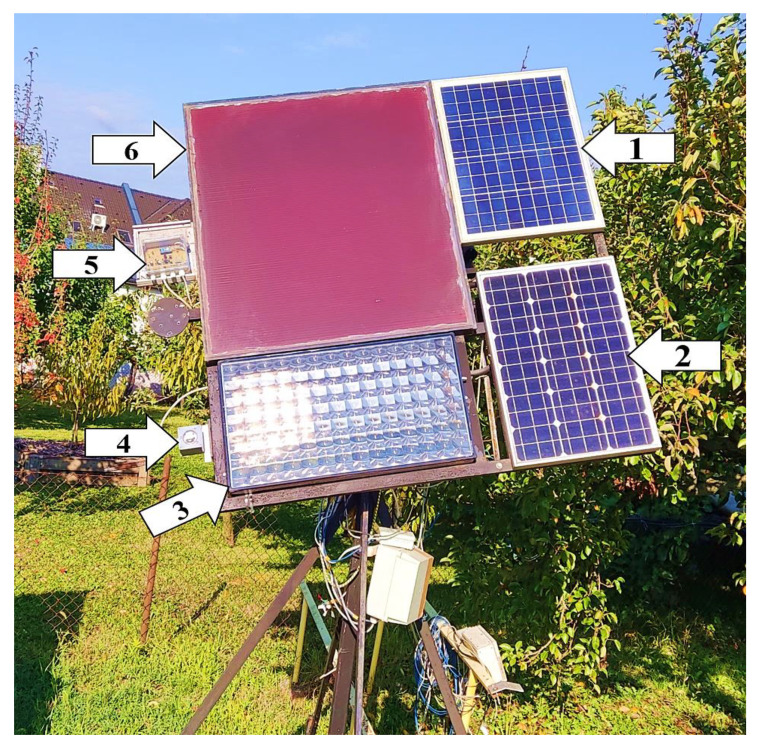
The measuring station in Keszthely: (1) p-Si module, (2) m-Si module, (3) CPV module, (4) LDR unit, (5) control unit with microcontroller, spatial position sensing unit, and luminous flux detection, (6) a-Si module.

The research was carried out at the following location: Hungary, Keszthely (northern hemisphere, latitude: 46.76750°, longitude: 17.26609°). The technical implementation of the tests involved a solar-tracking measuring station using a dual-axis rotation system with an m-Si, an p-Si, an a-Si, and a CPV module mounted on it (Figure 1).

**Figure 2:**
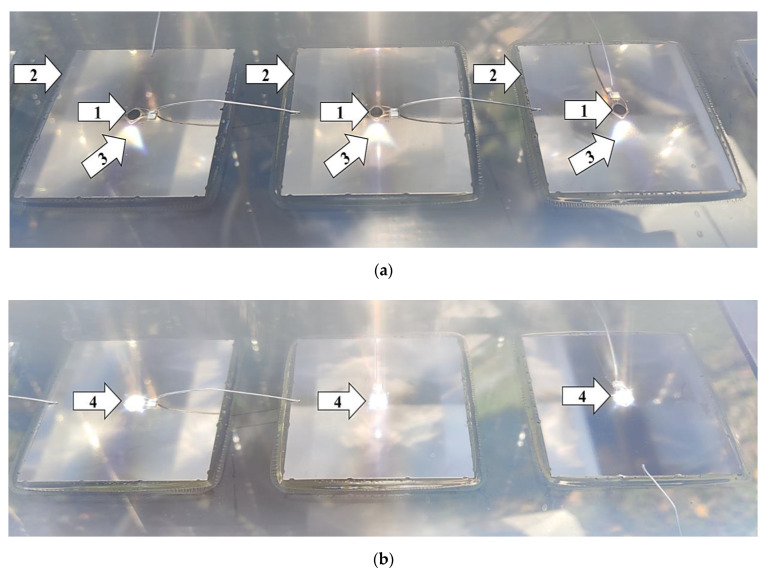
Illustration of the accuracy of solar tracking by using CPV technology (Image (**a**): (1) solar cell, Ø = 3 mm; (2) heat sink; (3) direct, focused solar radiation (focal point [61]) set incorrectly; Image (**b**): (4) direct, focused solar radiation (focal point [61]) set precisely).

One of the characteristics of the measuring station was that it was suitable for fitting/receiving any control and sensor unit capable of tracking the sun, so it allowed for such technologies to be tested. It was characterized by having (yes/no) input ports corresponding to the north–south (tilt angle) and east–west (azimuth angle) directions, with the help of which, depending on the signals received, the appropriate directions of movement could be controlled, and the specific operational characteristics of the technologies tested could be observed. The PV modules were moved by two linear actuators. The design of the system allowed both manual and automatic control, and its linear actuators could be switched on and off separately. With this solution, it was possible to examine not only the single- but also the dual-axis characteristics of solar tracking. During automatic use, both continuous and control unit-specific solar-tracking operations were possible. If manual control was used, the automatic sun-tracking function was disabled using a switch. The direct radiation visible inside the CPV module could be used to verify precise positioning towards the sun (Figure 2). In the FP position, the rays of the sun were ideally positioned and reached the cells of the CPV module perpendicularly (Figure 2b). The microcontroller, together with the devices necessary for controlling (Section 4), were mounted on the dual-axis rotating system (Figure 1).

The accuracy of solar tracking in the newly developed method was always determined by comparing it to the FP. Any inaccurate positioning was established by using digital protractors. The final version of the developed procedure was preceded by a 30-day test period, during which, deviations from the FP were constantly monitored, and with the help of the experience gained during operation, the program in the microcontroller was continuously improved.

## 4. The Results of the Development Project and the Novelty of the New Active Solar-Tracking Solution

### 4.1. The Theoretical Background of Developing the LDR Microcontroller Method

Theoretically, the direct radiation absorbed by a given surface is proportional to the cosine of the angle of incidence, while diffuse radiation is mainly influenced by the view factor between the surface and the sky [62]. In the case of direct radiation, an inaccuracy of up to 3°, therefore, results in a slight reduction in radiation of up to 0.14% (since cos(3°)=0.9986), while in the case of diffuse radiation, the effect of inaccuracy can be either positive or negative and, on average, typically negligible. In practice, the loss occurring due to inaccuracies of up to 3° is so small that a research based on several years of measurements was not able to detect a noticeable reduction in performance [46]. The range of 3° can, therefore, be considered as a point of insensitivity in the case of the most commonly used PV technologies, such as m-Si, p-Si, and thin film (e.g., a-Si), within which there is no loss of power during the operation of the PV modules, regardless of the direction of deviation and the technology [46]. The design of the LDR microcontroller presented in this paper is based on the recognition that if the above-mentioned range is used in solar tracking, there will be no noticeable decrease in the amount of electricity produced.

The solution introduced herein aims to address the identified shortcomings of the solutions described in Section 2. The benefits of this procedure include the following:

- Instead of constantly searching for the brightest point in the sky, periodic sun tracking is used to avoid the increased wear of the solar-tracking motors.
- In cloudy weather or heterogeneous light conditions, the given position gets fixed after searching for the brightest point in the sky for a maximum of 20 s. The maximum length of this period was determined based on experience from empirical observation. The duration of search is—of course—shorter if the brightest spot in the sky is found sooner, in which case, the search terminates.
- The procedure eliminates the swaying motion resulting from the continuous search for the brightest point in the sky, thus significantly reducing the risk of extra load on the motors and the deviation of the PV modules from the ideal position.
- A position that is favorable at sunrise is assumed, thusly assisting the PV system to instantly detect the sun’s rays at sunrise, so that it can reach an ideal position earlier than the solutions that keep the position they were in at sunset.
- Without an anemometer, it is possible to significantly reduce the damaging effects of strong winds during a storm as a result of setting the modules into an Equator-facing position with a tilt angle of 30° (0° azimuth and 30° tilt angle in the northern hemisphere). In comparison, in the case of an eastern or western tilt angle of more than 50°, the modules practically catch the wind just like sails and, in case of weaker mechanics, their support structures may be permanently damaged. The protection is based on the fact that during storms, the value of ambient illumination is less than 200 lux [63], which is why an Equator-facing position with a tilt angle of 30° is assumed according to the procedure. Furthermore, the flatter 30° inclination is more favorable in terms of collected radiation than a steeper tilt angle, as in overcast weather, a significant part of the diffuse radiation arrives near the zenith [64].
- During a storm, it is possible to protect the electronics and motors installed under the support structure against precipitation by assuming an Equator-facing position with a tilt angle of 30°. In the event of precipitation of great intensity, a small amount of water may enter the inside of the motor, increasing the likelihood of motor failure. For example, in the case of motors with IP 54 protection, some minimal leaking is tolerated, but in the longer term, it may cause failure in the electronics. Again, the protection is based on the fact that during storms, the value of illumination is below 200 lux, due to which, an Equator-facing position with a tilt angle of 30° is assumed according to the procedure.

### 4.2. The Components and Structure of the Newly Developed Solution

**Figure 3:**
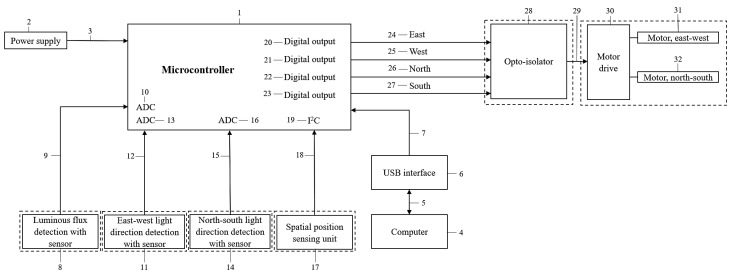
Schematic diagram of the components and structure of the newly developed solution.

**Figure 4:**
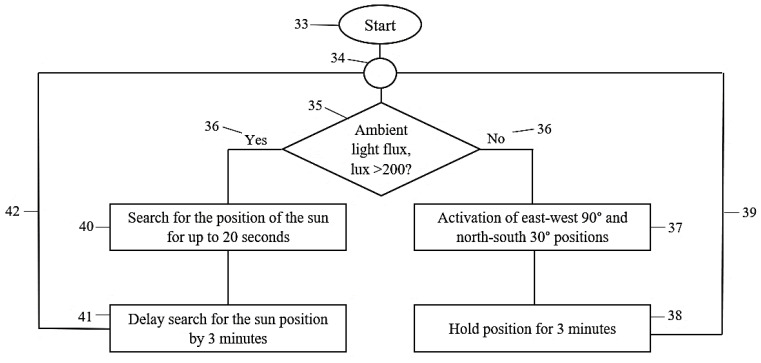
The characteristics of the logical operation of the newly developed method.

**Figure 5:**
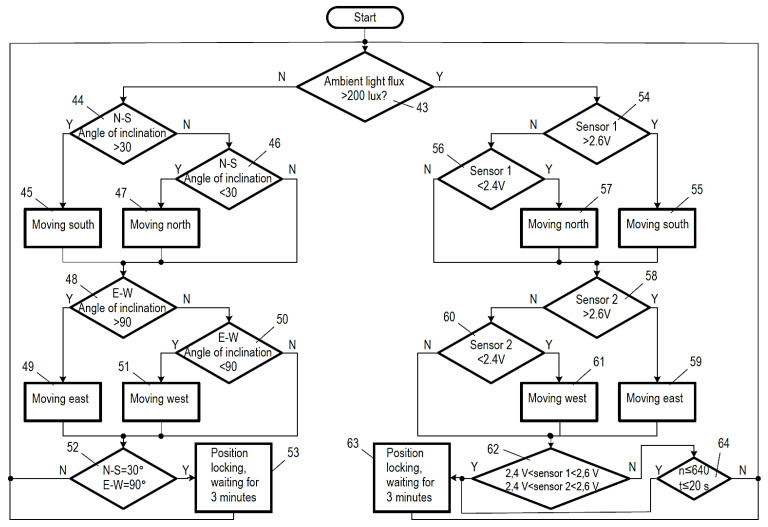
The characteristics of the detailed logical operation of the newly developed method.

The components and their relationships necessary for the operation of the newly developed method are explained in Figure 3. The numbering was added to facilitate comprehension:

- The microcontroller (1) operates with a stable power supply of 5V, which is provided by a built-in power supply (2) using a 10V network safety adapter (plug-in power supply unit), connected to the control unit by a waterproof bipolar connector (3).
- Any control modification, repair, or monitoring of the microcontroller can be done using a computer (4).
- The microcontroller and the computer can communicate (5, 7) via the USB interface (6).
- The sensor detecting ambient luminous flux (8) sends an analogue signal of 0 to 5V (9) to one of the analogue (ADC) (10) inputs of the microcontroller.
- The sensors that detect the east–west (11) and north–south (14) light directions also send analogue signals of 0 to 5V (12, 15) to an analogue input of the microcontroller (13, 16).
- The spatial position sensing unit (17) sends spatial data (18) to the microcontroller via the I2C communication port (19).
- Depending on the signals coming in from the sensors, the control strategies according to Figure 4 and Figure 5 run in the microcontroller, and at the four digital outputs (20, 21, 22, and 23) high-level signals (24, 25, 26, and 27) control the opto-isolators (28).
- The outputs of the opto-isolators are connected (29) to the motor-driving electronics (30), and then to the motors responsible for the north–south (31) and east–west (32) movements.

### 4.3. The Characteristics of the Logical Operation of the Newly Developed Method

The operation of control by the new solution is illustrated in Figure 4, which helps to understand the processes by numbering:

- During the operation of the system (33), several logical connections (34) run in one control strategy.
- First, an LDR sensor is used to determine the ambient luminous flux (lux) value (35).
- Then, yes/no logic based on the luminous flux (36) with a threshold of 200 lux is selected. That means that the appropriate logic in this example is determined by the measured level of illumination, which is either below or above the threshold set at 200 lux.
- In the event that the ambient light conditions have been significantly reduced or darkening occurs, the no process (36) is activated.
  ○ In this case, a value below 200 lux is detected, and the east–west 90° and north–south 30° position (37) (0° azimuth and 30° tilt angle) is activated, i.e., the sensors detecting sunlight assume a position that will allow the detection of sunrise.
  ○ The rotating motors move the sun-tracking mechanism into this position, and then, the position is secured (38).
  ○ The control reassesses the environmental characteristics every 3 min (39) and keeps this state until the luminous flux value exceeds 200 lux.
- In the event that solar radiation is detected, the yes procedure (36) is activated.
  ○ The control then searches for the brightest point in the sky (40) for up to 20 s, which is long enough according to empirical observation but also eliminates the superfluous operation of the solar-tracking motors in heterogeneous light conditions. In addition, (40) the constant swaying ensuing as a result of the continuous search for the bright spots of the sky is eliminated and, at the same time, the extra load on the motors and the risk of the deviation of the PV modules from the ideal position is also significantly reduced.
  ○ After finding the brightest point in the sky, the ideal position (41) is maintained for 3 min. Thus, instead of constantly searching for solar radiation, periodic solar tracking is performed every 3 min (without any loss of PV module power) to avoid the extra wear of the solar-tracking motors.
  ○ The control process reassesses the environmental characteristics every 3 min (42), and the yes procedure continues until the luminous flux value exceeds 200 lux.
- The 3 min intervals can also be set within a wider range, e.g., between 2 and 4 min; however, it has been taken into account in determining the 3 min length used in the procedure that, on the one hand, during this time, the change in the position of the sun at the research site in Hungary (and typically in the European countries in general) does not exceed 3° and, on the other hand, ambient light conditions can be temporarily affected by a number of events, such as accidental temporary cover, or the most likely event, passing clouds.

Figure 5 shows the more detailed logical operational aspects presented in Figure 4. Here, too, numbering helps understand the processes:

- After the system starts (start), the program runs continuously and restarts (resets) in the case of an error.
- Based on the amount of luminous flux, the signal generated by the sensor that detects ambient light (43) determines which control path is implemented; if the value of the ambient illumination is greater than 200 lux, the system continues on the yes control path, while in the case of a value of less than 200 lux, it continues on the no control path.
- In the case of a no command, the spatial position sensing unit determines if the north–south inclination is greater than 30° (44).
  ○ In the event that the north–south tilt angle of the PV module is greater than 30°, a southward (45) movement is triggered by a high-level signal from the digital output of the microcontroller (23) to the corresponding input of the opto-isolator, which causes the motor (32) to tilt the PV module in such a direction that reduces its north–south inclination.
  ○ If it is found that the north–south inclination of the PV module is not greater than 30°, the system proceeds to point 46, and it examines if the north–south inclination of the PV module is less than 30°.
  ○ If it is established in point 46 that the north–south inclination of the PV module is less than 30°, a northward (47) movement is triggered by a high-level signal from the digital output of the microcontroller (22) to the corresponding input of the opto-isolator, which causes the motor (32) to tilt the PV module in such a direction that increases its north–south inclination.
  ○ If in point 46 the north–south inclination of the PV module is not less than 30°, it means that the angle of inclination is equal to 30° south, and in this case, no high-level signal is emitted from the digital outputs of the microcontroller, so the motor does not start operation and does not move the PV module in either direction.
  ○ After points 45, 46, and 47, the process moves on to point 48, where it is examined if the east–west inclination of the PV module is greater than 90°.
  ○ In the event that the east–west angle of inclination of the PV module is greater than 90°, an eastward movement is carried out in point 49 by issuing a high-level signal from the digital output of the microcontroller (20), and then, this signal travels to the corresponding input of the opto-isolator, which causes the motor to operate and move the PV module in a direction that reduces the east–west inclination.
  ○ When it is established in step 48 that the east–west inclination of the PV module is not greater than 90°, the procedure moves on to point 50, and the procedure determines if the east–west inclination of the PV module is less than 90°.
  ○ In the event that point 50 establishes that the east–west inclination of the PV module is less than 90°, a westward movement is performed in step 51 by a high-level signal passing from the output of the microcontroller (21) to the corresponding input of the opto-isolator, which triggers the operation of the motor (31) to tilt the PV module in a way that increases its east–west inclination.
  ○ However, when it is determined in point 50 that the east–west inclination of the PV module is not less than 90°, it means that the angle of inclination is equal to 90°. In this case, outputs 20 and 21 of the microcontroller do not emit a high-level signal, so the motor does not start and does not tilt the PV module in either direction.
  ○ Points 49, 50, and 51 are followed by step 52, a further examination of whether the north–south inclination of the PV module is equal to 30° and the east–west inclination is equal to 90° or not. If there is a negative answer in either of the cases, the procedure goes back to point 43, and the described operation is reiterated.
  ○ When it is established in point 52 that the north–south inclination of the PV module is equal to 30° south and the east–west inclination equals 90°, the system concludes that the PV module is positioned correctly, and it is only the ambient illumination that is insufficient. In this situation, the given position is fixed in point 53, and a waiting period of 3 min commences, and it is only after that when the procedure returns to step 43 to repeat the described operation.
- On the yes control path, in point 54, it is determined whether the output voltage of the sensor detecting the north–south illumination is higher than 2.6 V, less than 2.4 V, or if it falls between the two values.
  ○ If the output voltage of the sensor detecting the north–south illumination is higher than 2.6 V, a southward movement is carried out in point 55, triggered by a high-level signal from the digital output of the microcontroller (23) to the corresponding input of the opto-isolator, which starts the operation of the motor (32), moving the PV module in the suitable direction, which reduces the north–south inclination.
  ○ When in point 54 it is found that the output voltage of the sensor responsible for the north–south light is not greater than 2.6 V, the procedure moves on to step 56 and examines if the output voltage of the sensor is less than 2.4 V.
  ○ If in point 56 it is established that the output voltage of the sensor detecting the north–south illumination is lower than 2.4 V, a northward movement is carried out in point 57, triggered by a high-level signal from the digital output of the microcontroller (22) to the corresponding input of the opto-isolator, which starts the operation of the motor (32), tilting the PV module in a way that prevents the north–south inclination of the PV module from increasing.
  ○ When in point 56 it is found that the output voltage of the sensor responsible for the north–south light is not less than 2.4 V, it means that the tilt angle is appropriate. In such a case, outputs 22 and 23 of the microcontroller do not emit a high-level signal, so the motor does not start and does not tilt the PV module in either direction.
  ○ After points 55, 56, and 57 alike, the procedure moves on to point 58, in which it is verified if the output voltage of the east–west illumination sensor is greater than 2.6 V, less than 2.4 V or between the two values.
  ○ If the output voltage of the sensor detecting the east–west illumination is higher than 2.6 V, an eastward movement is performed in point 59, triggered by a high-level signal from the digital output of the microcontroller (20) to the corresponding input of the opto-isolator, which starts the operation of the motor (31), moving the PV module in the suitable direction, which reduces the east–west inclination.
  ○ When in point 58 it is found that the output voltage of the sensor responsible for the east–west light is not greater than 2.6 V, the procedure moves on to step 60 and examines if the output voltage of the sensor is less than 2.4 V.
  ○ If in point 60 it is determined that the output voltage of the sensor detecting the east–west light is less than 2.4 V, then in point 61, a westward movement is performed by a high-level signal passing from the output of the microcontroller (21) to the corresponding input of the opto-isolator, which makes the motor start and tilt the PV module in such a direction that increases its east–west inclination.
  ○ If, however, in point 60, it is found that the output voltage of the sensor detecting east–west light is not less than 2.4 V, that means that the angle of inclination is within the acceptable range. In this case, outputs 20 and 21 of the microcontroller do not emit a high-level signal, so the motor does not start and does not tilt the PV module in either direction.
  ○ After points 59, 60, and 61 alike, the procedure moves on to point 62, in which it is checked if the output voltages of the sensors detecting the north–south and the east–west illumination are both within the 2.4–2.6 V range.
  ○ If it is established in step 62 that the output voltages of the sensors sensing the north–south and the east–west light directions are both in the range of 2.4–2.6 V, it means that the PV module is in an appropriate position.
  ○ In this case, the position is fixed in step 63 and a waiting period of 3 min ensues, and it is only after that when the procedure returns to point 43 to reiterate the described operation.
  ○ In the event of a negative response in any of the cases, the check is repeated for a maximum of 20 s and no more than 640 times, which is verified in point 64, and if the 640 searches are unsuccessful or the 20 s have elapsed, the position is maintained in point 63 and the 3 minutes’ waiting time ensues, and then the procedure goes back to step 43 and reiterates the described actions.

The average inaccuracy of the final version of the developed method was 1.9° at the end of the 30-day testing period, which means that solar tracking without visible performance loss is feasible.

## 5. Discussion

The sun-tracking solutions available on the market are either simple or more sophisticated, specific constructions. The simpler solutions are inaccurate; they are not able to locate the FP, and usually, they do not put the PV modules in the ideal dawn position after sunset or at sunrise, so the sensors that detect sunlight can only do so later, after a significant loss of time. This has a negative effect on daily energy production. Another common problem with simpler solutions is the constant search for the brightest spot in the sky, which causes a constant search for direction in cloudy weather. This means extra loads and faster, premature wear for the motors responsible for solar tracking, which practically results in less generated energy, since the unnecessary operation of the motors causes extra energy uptake. The specific constructions are primarily designed for CPV systems, in which the FP is continuously maintained. Unlike simpler constructions, these are capable of precise solar tracking, but they do not provide a suitable solution to several problems (e.g., the constant, fine motor movements for precision).

In summary, it can be concluded that nowadays, in the case of solar-tracking PV systems based on active sensor driver systems, no control mechanism is available that possesses all of the following features: a periodic search for the brightest point in the sky; the prevention of superfluous motor activity due to the search for position resulting from cloudiness; setting the system at sunset to a position ideal at sunrise in the following morning; the significant reduction in the damaging effects of wind generated during a storm and the joint protection of the electronics and motors installed under the support structure against getting wet without the use of an anemometer.

The construction presented in this study eliminates the above shortcomings in a complex way. Instead of constantly searching for the brightest point in the sky, the system performs periodic solar tracking (the given position is maintained for 3 min, and after repositioning, it blocks the movement of the sun-tracking motors again for another 3 min), thus preventing extra mechanical wear. In cloudy weather or heterogeneous light conditions, it secures the position after searching for the brightest point in the sky for up to 20 s, which is long enough according to empirical observation but also eliminates the continuous swaying movement resulting from the constant search for the brightest spot in the sky. At sunrise, it helps to take an advantageous position so that the system can immediately detect the appearing rays of the sun. Without an anemometer, it is capable of significantly reducing the damaging effects of wind during a storm by assuming a southward position. During a storm, it provides protection for the electronics and motors installed under the support structure against getting wet by adopting the southward position.

For practical applications, the newly developed algorithm effectively avoids unfavorable positions (especially around sunrise and sunset), thus increasing expected energy production despite a tracking error of a few degrees. The method also reduces the wear of the motors resulting from wind load and swaying movements, thus also decreasing maintenance costs. The most favorable financial return on PV parks can be ensured by the optimal balance of production and costs, so the practical application of the developed method clearly contributes to improving their profitability. As the majority of the life-cycle environmental impacts of a PV park come from the PV modules [65], maximizing the energy generation of the modules also contributes to reducing the environmental impacts.

## 6. Conclusions

In densely populated countries, the power of PV systems per unit area is especially crucial, as the land available for building PV systems is limited. Consequently, increasing the yearly specific yield is a priority, which is attainable by deploying single and dual-axis solar-tracking solutions, capable of helping to reach an increase of even 20–45%. Moreover, besides a great solar-tracking performance, a useful solar-tracking solution is also expected to boast a long lifetime and low maintenance costs. Additionally, it is this particular aspect in which today’s common active sensor tracking solutions demonstrate their greatest limitations due to their failure to prevent the unnecessary operation of the motors when the sky is cloudy and to protect the structure and the electronics of the PV systems from the elements in the event of a storm. Furthermore, most of the algorithms in use today simply leave the modules in a position facing west after sundown, leading to a waste of time and a loss of energy resulting from a delay in starting to generate power after daybreak.

All the problems outlined above can be solved by the newly developed algorithm introduced in this paper, which primarily makes use of the finding that the most commonly used PV technologies have a so-called insensitivity threshold of inaccuracy of 3°. This means that regardless of the direction of the deviation and the technology, no power loss occurs resulting from a deviation of up to 3° during PV operation. Relying on this, the presented solution deploys periodic tracking with a 3 min delay, as during this time, the change in the position of the sun is typically below 3°. Additionally, the algorithm described herein moves the modules into a position facing the equator, with a tilt angle of 30°, whenever the illumination is less than 200 lux. This is done in order to achieve the double goal of ensuring optimal positioning at sundown and the protection of the equipment from rain and winds during storms, without using an anemometer. The single control procedure of the new solution was created on the basis of the ideas above to attain the objectives of maintaining position in cloudy weather conditions to prevent the continuous search for the brightest spot in the sky, and thus solve the problem of the unnecessary swaying motion of the motors; the significant reduction in the risk of damage by strong winds in the event of a storm; the protection of the electronics and the motors under the support structures from the rain; the appropriate positioning of the system in the event of inadequate light and at sunset to facilitate start up when light conditions change.

A further aim of the research introduced herein is to develop, based on an active sensor driver system, a modularly adaptable cloud detection unit and sensor for solar-tracking systems that are capable of generating control signals for solar radiation, clouding, and ambient light conditions.

## Figures and Tables

**Table 1:** Summary of the characteristics of the control mechanisms of the solar-tracking solutions using active sensor driver systems reviewed above.

Solar-Tracking Solutions	Seeking Brightest Point in the Sky		Protection against Clouding	Protection against the Unnecessary Swaying of Motors	Protection against Wind	Assuming Position after Sunset
	Continuous	Periodic				
FUSIONSEEKER, DS-50D6W and DS-100D10 [50]	●	-	○	○	○	●
ECO-WORTHY, dual-axis solar tracker controller [51]	●	-	○	○	○	●
WST03-2 [52]	●	-	-	-	○	-
Luoyang Longda Bearing Co., Ltd., solar-tracking controller [53]	●	-	-	-	○	-
SunTura solar tracker [54]	●	-	-	-	-	-
STA2000-HW [55]	●	●	○	○	●	○
MLD sensor [56,57]	●	-	-	-	-	●
WO 2020/185271 A1 patent description [58]	●	-	○	○	●	●
Patent description number EP 2 593 759 B1 [59]	●	-	○	○	-	●
Patent description WO 2013/074805 A1 [60]	●	-	-	-	-	-
The method presented in this paper	○	●	●	●	○	●

The ●/○/- marks mean yes/partially or another device is required for operation/no.

## Data Availability

The data presented in this study are available within the article.

## Abbreviations

The following abbreviations are used in this manuscript:

AI	artificial intelligence
ANFIS	adaptive neural fuzzy inference systems
a-Si	amorphous silicon
CPV	concentrator photovoltaic
CSP	concentrating solar power
DIL	dual in-line
EU	European Union
FNN	fuzzy neural network
FP	focal point
FREN	fuzzy rules emulated network
LDR	light dependent resistor
m-Si	monocrystalline silicon
PIC	programmable interface controller
p-Si	polycrystalline silicon
PV	photovoltaic
TMPS	true maximum point seeking
